# Preictal Dynamics of EEG Complexity in Intracranially Recorded Epileptic Seizure: A Case Report

**DOI:** 10.1097/MD.0000000000000151

**Published:** 2014-11-14

**Authors:** Petr Bob, Robert Roman, Miroslav Svetlak, Miloslav Kukleta, Jan Chladek, Milan Brazdil

**Affiliations:** Central European Institute of Technology (PB, RR, MK, JC, MB); Department of Physiology (RR, MS, MK); Department of Neurology (MB), Faculty of Medicine, Masaryk University, Brno; Center for Neuropsychiatric Research of Traumatic Stress (PB, MS, JC), Department of Psychiatry and UHSL, 1st Faculty of Medicine, Charles University, Prague; and Institute of Scientific Instruments, Academy of Sciences of the Czech Republic, Brno, Czech Republic.

## Abstract

Recent findings suggest that neural complexity reflecting a number of independent processes in the brain may characterize typical changes during epileptic seizures and may enable to describe preictal dynamics. With respect to previously reported findings suggesting specific changes in neural complexity during preictal period, we have used measure of pointwise correlation dimension (PD2) as a sensitive indicator of nonstationary changes in complexity of the electroencephalogram (EEG) signal. Although this measure of complexity in epileptic patients was previously reported by Feucht et al (Applications of correlation dimension and pointwise dimension for non-linear topographical analysis of focal onset seizures. *Med Biol Comput*. 1999;37:208–217), it was not used to study changes in preictal dynamics. With this aim to study preictal changes of EEG complexity, we have examined signals from 11 multicontact depth (intracerebral) EEG electrodes located in 108 cortical and subcortical brain sites, and from 3 scalp EEG electrodes in a patient with intractable epilepsy, who underwent preoperative evaluation before epilepsy surgery. From those 108 EEG contacts, records related to 44 electrode contacts implanted into lesional structures and white matter were not included into the experimental analysis.

The results show that in comparison to interictal period (at about 8–6 minutes before seizure onset), there was a statistically significant decrease in PD2 complexity in the preictal period at about 2 minutes before seizure onset in all 64 intracranial channels localized in various brain sites that were included into the analysis and in 3 scalp EEG channels as well. Presented results suggest that using PD2 in EEG analysis may have significant implications for research of preictal dynamics and prediction of epileptic seizures.

## INTRODUCTION

According to current findings, dynamic changes in the brain during epileptic seizures may be described using measures of neural complexity that reflect transitions in neural networks that may be assessed using nonlinear time-series analysis.^1,2^ The concept of neural complexity is based on mathematical description that enables to assess whether activities of a neural system are dynamically segregated, which means that small subsets of the system may tend to behave independently or they are dynamically integrated and manifest coherent and synchronous activity.^3,4^ These changes in dynamical complexity of the neural systems may describe the dynamic equilibrium between differentiation and integration in the complex neuronal dynamics and its development during the time.^3,5^

Together, these data suggest that increased neural complexity calculated from the electroencephalogram (EEG) and other psychophysiological measures may reflect processes related to increased activity of independent areas that enable desynchronized parallel information processing, and on the contrary, decreased complexity is related to synchronization phenomena in the brain that typically occurs during epileptic seizures.^2,6,7^ These data may explain why preictal dynamics may lead to changes in neural complexity preceding epileptic seizure as several studies suggest.^2,8–11^

With respect to current findings, the main purpose of this case study is to analyze the preictal dynamics of the EEG complexity, assessed using algorithm for pointwise correlation dimension (PD2), focused to find differences in the EEG complexity characterizing the preictal period. Although the PD2 as a sensitive measure of nonstationary changes of complexity in epileptic patients was reported by Feucht et al,^8^ to the best of our knowledge, it was not used to study changes in the complexity of preictal dynamics. In this context, using this sensitive measure of complexity may help to resolve some contradictory results obtained using various nonlinear measures^9^ and quantify changes in complexity during the preictal period more precisely in comparison to other nonlinear indices.

## METHODS

### Participant

Twenty-four-year-old right-handed man suffering from intractable extratemporal epilepsy because of suspected multifocal tuberous sclerosis participated in the study. The patient manifested frequent complex partial seizures with initial loss of consciousness, vocalizations, and late right-hand automatisms but without aura. For presurgical evaluation, 11 orthogonal standard MicroDeep multilead depth electrodes (ALCIS, Besançon, France), with a diameter of 0.8 mm, length of each recording contact 2 mm, and intercontact intervals of 1.5 mm, were used for invasive EEG monitoring. The electrodes were implanted in the frontal, temporal, occipital, and parietal lobes using the methodology by Talairach et al^12^ and placed bilaterally. The exact positions of the electrodes in the brain were verified using post-placement magnetic resonance imaging with electrodes in situ. Both the number and positions of these electrodes were determined by a diagnostic procedure with the aim to localize the seizure origin prior to a surgical treatment. The recordings from 44 channels implanted into lesional structures and white matter were not included into the experimental analysis. In addition to intracranial recordings, the EEG measure also included 3 scalp electrodes (Fz, Cz, and Pz). In this presurgery evaluation, the epileptogenic zone most likely responsible for generation of epileptic activity was located in the middle frontal gyrus.

With the aim to study preictal changes of complexity from these electrodes, we have analyzed EEG records from 64 intracranial channels, localized in 11 brain regions, and 3 scalp channels. Prior to the experiment, the patient provided informed consent, and the study was approved by the Ethical Committee, Masaryk University, Brno, Czech Republic.

### EEG Recording

The EEG signal was simultaneously recorded from various intracerebral structures and from standard Fz, Cz, and Pz scalp electrodes, using the 128-channel TrueScan EEG system (Deymed Diagnostic, Payette, ID). All recordings were monopolar with earlobe reference. All impedances were <5 kΩ. The sampling rate was 1 kHz.

The long-term video-EEG recording was aimed to distinguish preictal (before), ictal (during), and postictal recordings (after the clinical seizure). The clinical and electroencephalographic seizure onset was reviewed by 2 experienced clinical epileptologists. The EEG showed 1 seizure but more quasi-independent partial seizures cannot be rejected. Partial seizure was not confirmed and hidden multiple foci cannot be rejected.

From these EEG records, 8-minute-long records before electroencephalographic seizure onset, 2-minute-long records during the seizure, and 8-minute-long records after the seizure were used. For the analysis and statistical comparison, 2-minute-long records were used, 4 from the preictal period, 1 during seizure, and 4 after the seizure.

### Data Analysis

A practical approach to studying complex dynamical systems is the method of time-series analysis. A postulate of this method is that every dynamic system, for example, the human brain and its functions, is governed and determined by a number of independent variables.

The time-series analysis therefore represents a mathematical approximation that enables a reconstruction of certain variables underlying the multidimensional dynamics from data obtained from the system during the time,^13^ for example, a psychophysiological measurement performed on the system during an experiment. Because observational data reflect only a few independent variables of the system, approximation of the dynamic system behavior therefore uses a finite number of (mathematically reconstructed) variables to approximate states of the system. The multidimensionality of the dynamic system is therefore approximated by an embedding dimension that represents its dimensionality contained (or embedded) in the data.

In the analysis, the 2-minute-long EEG records characterizing brain activity during experimental periods were processed using the software package Dataplore that is one of the most well-known software implementations for time-series analysis. Skinner algorithm for the PD2 was used in the analysis.^14^

The PD2 algorithm for this calculation was selected because of its usefulness to detect the complexity of phase transitions in nonstationary time series of dynamic systems.^13^ Calculation of the PD2 is based on the formula PD2(*i*) = logC(*r*,*i*)/log(*r*), where C(*r*,*i*) is pointwise correlation integral that gives a average sort time over the whole signal that provides a value for each point in time of the signal and *r* is a distance reflecting radius of the neighborhood around a certain point (between 0 and 1).

All PD2 calculations were separately performed for each EEG channel (64 intracranial and 3 scalp electrodes) on the raw data with parameters for embedding dimension from 2 to 8. These parameters are typical for majority of EEG signals and enable rigorous replication. Following it, statistical evaluation of PD2 values for the preictal, ictal, and postictal periods was performed using *t* test by software package Dataplore (*t* test is for high *df* values equivalent with nonparametric tests). Further data analysis was performed using descriptive statistics (means and standard deviations), Wilcoxon test, and Friedman ANOVA using software Statistica version 8.0 (StatSoft, Inc, Tulsa, OK).

## RESULTS

**FIGURE 1:**
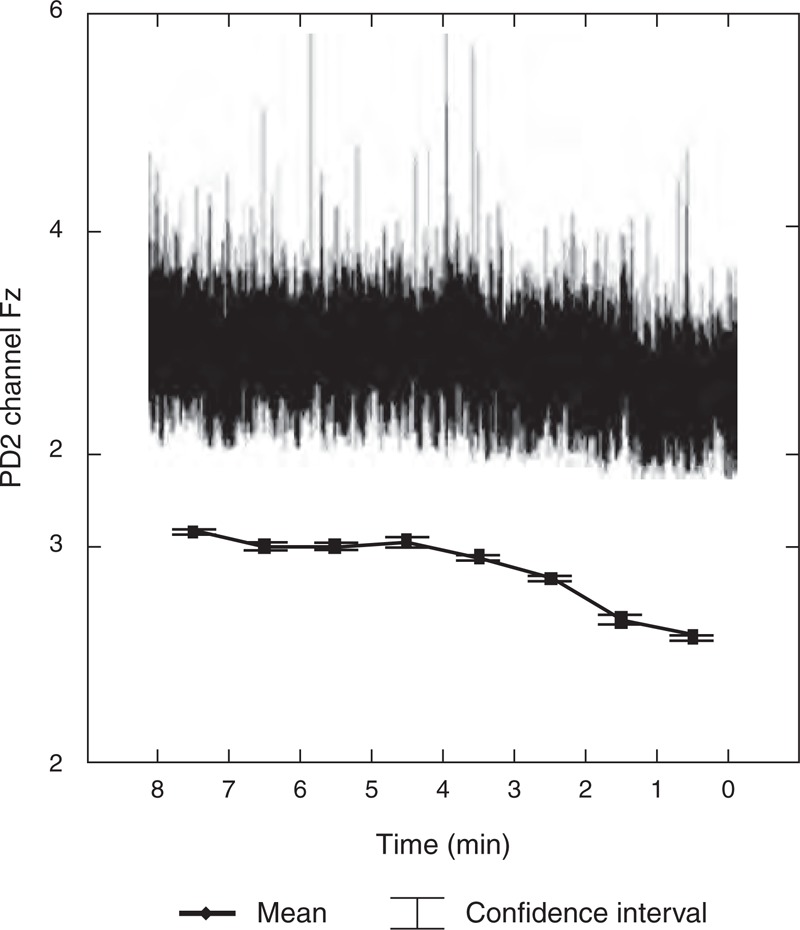
Time dependence of complexity (PD2) in channel Fz during preictal period (8-0 min. before seizure) that has the maximum difference between first and fourth preictal periods.

The results show that in all 64 contacts localized in 11 subcortical sites and 3 scalp channels, statistically significant decrease in PD2 complexity between 1st and 4th preictal period has been found. The data also show significant decrease in PD2 complexity between preictal and ictal period and statistically significant increase in PD2 complexity between ictal and postictal period (Table 1). Topographical analysis focused on maximum differences in the EEG complexity indicator (PD2) characterizing preictal dynamics of EEG complexity shows that very high differences between 1st and 4th preictal period were found in frontal operculum and insula, scalp channel Cz, and the highest difference was found in scalp region Fz (Table 1, Figure 1).

**TABLE 1:**
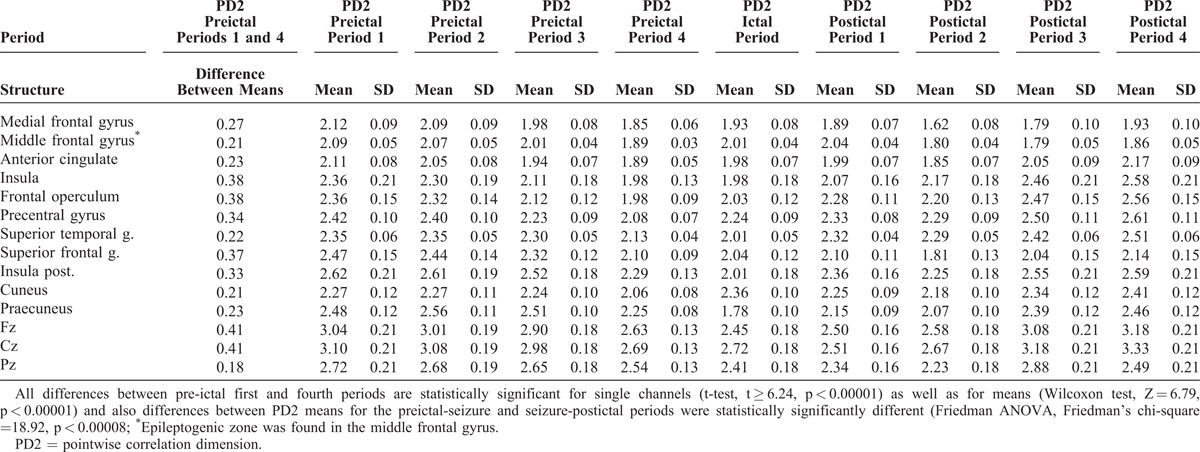
Values of Complexity (PD2) in the Brain Implanted and Scalp Electrodes During Preictal, Ictal, and Postictal Periods

In summary, the results show significant differences between preictal, ictal, and postictal periods in all investigated brain sites. In the preictal periods, synchronized decrease in complexity was observed, which suggests that many distant parts of the brain were in abnormal synchronous state reflected by PD2, although descriptive EEG in the preictal period did not provide this information.

## DISCUSSION

The results of this case study indicate that EEG complexity measured by PD2 may represent useful and applicable indicator for description of preictal dynamics, and its significant decrease was observed in all analyzed implanted and surface electrodes used in presurgical EEG assessment. These characteristic changes describing preictal dynamics may likely be explained by synchronous widespread excitatory and inhibitory influences in distant brain regions preceding seizure activity that may be reflected in nonlinear dynamical description using complexity measures. In this context, various studies describing dimensional complexity and chaotic dynamics related to neural dynamics and EEG have been reported including those describing increased or decreased neural complexity and other nonlinear indicators preceding epileptic seizures.^2,8–11^ For example, Lehnertz and Elger^11^ found a focal decrease in complexity measured using correlation dimension D2 during epileptic events, and some other studies have found that complexity of the epileptogenic area can be influenced by anticonvulsant drugs.^15^ In addition, Feucht et al^8^ using correlation dimension and “pointwise dimension” in analysis of the surface EEG records of 8 patients have found that pointwise dimension was able to identify “strong epileptic activity” compared with control data. This identification of epileptic activity, in principle, is based on significant changes in neural complexity that distinguishes these regions from other brain sites.

Decreased or increased complexity described by various nonlinear indices likely reflects various levels of integration and disintegration that may occur in strongly coupled networks as well as weakly coupled cortical networks, both of which can create the same process of neural bursting and generate epileptic activity.^6^ In this context, data of the present study although limited and need further confirmation suggest that using PD2 measure may have significant implications for research of preictal dynamics and prediction of epileptic seizures.
